# Design of a Customized Multipurpose Nano-Enabled Implantable System for *In-Vivo* Theranostics

**DOI:** 10.3390/s141019275

**Published:** 2014-10-16

**Authors:** Esteve Juanola-Feliu, Pere Ll. Miribel-Català, Cristina Páez Avilés, Jordi Colomer-Farrarons, Manel González-Piñero, Josep Samitier

**Affiliations:** 1 Department of Electronics, Bioelectronics and Nanobioengineering Research Group (SIC-BIO), University of Barcelona, Martí i Franquès 1, Barcelona 08028, Spain; E-Mails: pmiribel@el.ub.edu (P.L.M.-C.); cpaezaviles@el.ub.edu (C.P.A.); jcolomer@el.ub.edu (J.C.-F.); jsamitier@ibecbarcelona.eu (J.S.); 2 Department of Public Economy, Political Economy and Spanish Economy, University of Barcelona, Av. Diagonal 690-696, Barcelona 08034, Spain; E-Mail: manel.gonzalez@ub.edu; 3 CREB-Biomedical Engineering Research Centre, Technical University of Catalonia, Pau Gargallo 5, Barcelona 08028, Spain; 4 IBEC-Institute for Bioengineering of Catalonia, Nanobioengineering Research Group, Baldiri Reixac 10-12, Barcelona 08028, Spain; 5 CIBER-BBN-Biomedical Research Networking Center in Bioengineering, Biomaterials and Nanomedicine, María de Luna 11, Edificio CEEI, Zaragoza 50018, Spain

**Keywords:** implantable multi-sensor, biosensor, biotelemetry, biocompatible, KET, nanomedicine, personalized medicine, innovation

## Abstract

The first part of this paper reviews the current development and key issues on implantable multi-sensor devices for *in vivo* theranostics. Afterwards, the authors propose an innovative biomedical multisensory system for *in vivo* biomarker monitoring that could be suitable for customized theranostics applications. At this point, findings suggest that cross-cutting Key Enabling Technologies (KETs) could improve the overall performance of the system given that the convergence of technologies in nanotechnology, biotechnology, micro&nanoelectronics and advanced materials permit the development of new medical devices of small dimensions, using biocompatible materials, and embedding reliable and targeted biosensors, high speed data communication, and even energy autonomy. Therefore, this article deals with new research and market challenges of implantable sensor devices, from the point of view of the pervasive system, and time-to-market. The remote clinical monitoring approach introduced in this paper could be based on an array of biosensors to extract information from the patient. A key contribution of the authors is that the general architecture introduced in this paper would require minor modifications for the final customized bio-implantable medical device.

## 1. Introduction

The current interaction between medicine and technology permits the development of new diagnostic devices to detect or monitor pathogens, ions, diseases, *etc.* Doubtless, the integration of rapid advances in areas such as microelectronics, microfluidics, microsensors and biocompatible materials entails the availability of implantable biodevices for continuous monitoring or event detectors that carry out faster and cheaper clinical tasks than when these are done by standard methods. Implantable devices have already been used in millions of patients [1]. Benefits of these approaches include improved care and quality of life [2]. Implantable sensor networks can facilitate an early detection of emergency conditions and diseases in patients at risk, [3] comprising physical, physiological, psychological, cognitive, and behavioral processes [4], by reaching inaccessible environments in a reduced response time [5].

It is in this context that we present a proposal of an integrated front-end architecture for *in vivo* customized detection. A new and challenging scenario defined as the pervasive system is focused on the development of systems capable of monitoring human bodily functions and to transmit the resultant data for a clinical patient's monitoring [6]. Thanks to this approach, it could be possible to monitor patients anywhere and at all times with important impact on their quality of healthcare preventing the worst scenarios for the patients as well as improving the wellbeing and continuing activity of the whole population. The possibility of controlling how a therapy is working, detecting symptoms, and knowing how the disease is progressing will improve the personalized medical care known as theranostics. Patients at risk because of their genetic background, chronically ill or elderly people will be monitored outside of and beyond visits to the hospital or at the surgery. Here, the significant advantage is to monitor patients in their routine daily activities, as traditional clinical monitoring would be replaced by continuous and remote monitoring [7]. This could have a great impact on patients' quality of life and could reduce the cost of the overall healthcare system [8]. Across all medical applications and diseases, findings suggest that chronic illness deserves special attention [9], particularly in the case of cardiovascular illness [10].

With the aim of medically monitoring the patients, there are two different approaches that are typically used: external body sensors, and implantable devices, *i.e.*, non-invasive approaches *versus* the invasive ones. In the case of external sensors for non-invasive physiological monitoring [11], a multiplatform is suggested [12], with particular interest in the wearable solutions and unobtrusive sensing methods [13,14] and, in particular, the most recent advances in textile-based electronics are relevant [15]. In the case of the invasive techniques that are the focus of this document, the type of solutions that have been developed, and those which are currently in progress [16], have as a classic example the cardiac implant, which was the initial application of these devices, now with advanced capabilities such as recently reported by Lee *et al.* [17]. The evolution of semiconductor technology, with low-voltage and low-power electronics, allows the integration of several implantable devices for different functions. These approaches could also be combined in order to define a body sensor network (BSNs) [18]. The placing of a central control node that acts as a master node, with other slave nodes located on or inside the body, monitoring different vital signals, defines a typical wireless network that could fulfil the theranostics needs of the patients.

Theranostics covers a wide range of applications as health interventions with drugs (pharmacogenomics), nutrition (nutrigenomics) and vaccines (vaccinomics), as well as diagnostics for human diseases [19]. Implantable medical devices are widely used for therapeutic [5] or life-saving purposes such as cardiac arrhythmia, diabetes, and Parkinson's disease [20]. Applications include drug delivery systems, pacemakers, implantable cardiac defibrillators (ICDs) and Neurostimulators [1]. Some real-time monitoring applications include physiological parameters like blood pressure, glucose levels and collecting data for further analysis [5].

These devices often contain electronic components that perform increasingly sophisticated sensing, computation, and actuation, in many cases without any patient interaction [1] as in the applications mentioned above, performing complex analyses with sophisticated decision-making capabilities. They are capable of storing detailed personal medical information, and communicate automatically, remotely, and wirelessly [2]. Implanted biosensors form a wireless network that can be used for data aggregation and data dissemination applications [5].

The system introduced in this paper is conceived to be implanted under the human skin. The powering and communication between this device and an external primary transmitter are based on an inductive link [21]. The design presents two different approaches: defining a true/false alarm system based on either amperometrics or impedance into a grid of nano-biosensors that could permit the monitoring of several diseases by *in vivo* analysis of the corresponding biomarkers.

## 2. Description and Challenges of a Customized Biomedical Implantable Device

### 2.1. State-of-the-Art of the Multipurpose Diagnosis Implantable Devices

Many different problems need to be overcome in obtaining the ideal implantable device [22]. First and foremost, the device must be biocompatible to avoid unfavourable reactions within the body. Secondly, the medical device must provide long-term stability, selectivity, calibration, miniaturization and repetition, as well as power in a downscaled and portable device. In terms of the sensors, label-free electrical biosensors are ideal candidates because of their low cost, low power and ease of miniaturization. Recent developments in nanobiosensors provide suitable technological solutions in the field of glucose monitoring [23,24], pregnancy and DNA testing [25], and microRNA detection [26]. Electrical measurement, when the target analyte is captured by the probe, can exploit voltometric, amperometric or impedance techniques. Ideally, the device should be able to detect not just one target agent or pathology, but rather several different agents and it should be capable of working in a closed-loop feedback, as described by Wang [27] in the case of glucose monitoring.

Several biomedical devices for *in vivo* monitoring are currently being developed [24,28,29]. Thus, highly stable, accurate intramuscular implantable biosensors for the simultaneous continuous monitoring of tissue lactate and glucose have recently been produced, including a complete electrochemical cell-on-a-chip. Moreover, with the parallel development of the on-chip potentiostat and signal processing, substantial progress has been made towards a wireless implantable glucose/lactate sensing biochip [30]. Elsewhere, implantable bio-micro-electromechanical systems (bio-MEMS) for the in situ monitoring of blood flow have been designed [31]. Here, the aim was to develop a smart wireless sensing unit for non-invasive early stenosis detection in heart bypass grafts. Interestingly, this study examines the use of surface coatings in relation to biocompatibility and the non-adhesion of blood platelets and other blood constituents. In this case, the nanotechnology as a KET seems to be a useful tool for improving the biocompatibility of silicon bio-MEMS structures.

A theranostic device has one or more specific molecular recognition markers for cells on the surface thereof, wherein the recognition markers are selected from the group consisting of peptides, proteins, antibodies, antigens, aptamers, molecular imprinted polymers and polynucleotides. When the device is implanted in a body, cellular ingrowth is controlled, with desired cell types anchoring and proliferating on the implant's surface to generate a thin layer, and thereafter ceasing accumulation. The cellular layer thereby presents a biomimetic surface acceptable to the body, and also presents a low barrier to diffusion of analytes with at least substantially constant diffusion characteristics, allowing the use of an analyte sensor within the article.

In biomedical research, there is a great need for multipurpose, reliable, and possibly implantable telemetric tools. By using sensor inputs, such devices allow the automated gathering of information on physiological parameters without restraining or stressing their subjects. For this purpose, a versatile implantable and four-channel telemetry data-acquisition unit was implemented by Wouters *et al.* [32], in a 2-pm n-well CMOS process. The dimensions of this single-chip implementation are 4.7 × 7.1 mm^2^. In the form of an implantable or portable telemetry system, a low-power mixed analog-digital CMOS integrated circuit combining several sensor interfaces, the processing and control circuitry, and the telemetry unit is intended for monitoring body temperature and physical activity.

A versatile theranostic system was developed by Young Choi *et al.* [33] for the early detection, targeted therapy, and therapeutic monitoring of colon cancer, by using poly(ethylene glycol)-conjugated hyaluronic acid nanoparticles (P-HA-NPs) which can selectively accumulate in tumor tissue. For the diagnostic application, a near-infrared fluorescence (NIRF) imaging dye (Cy 5.5) was chemically conjugated onto the HA backbone of P-HA-NPs. Arjang Hassibi has worked in the areas of biosensors and bioelectronics, biomedical electronics, and integrated circuit design. His company, InSilixa, is working on a multi-diagnostic system, using semiconductor-integrated DNA-sequencing technologies to create point-of-care diagnostics devices. The idea is to take advantage of “large-volume semiconductor technology,” manufacturing systems that are widely available and well established, to gain economies of scale [34].

An RF-powered wireless three-channel implantable bio-sensing microsystem has been developed with blood pressure, EKG, and core body temperature sensing capability for untethered genetic tests. A flat silicone blood pressure sensing cuff with a MEMS capacitive pressure sensor is employed to form a novel less-invasive blood pressure sensor, which avoids vessel occlusion, bleeding, and blood clotting associated with the conventional catheter-based sensors. The implantable microsystem can be powered by an adaptively controlled external RF energy source at 4 MHz to ensure a stable on-chip power supply. On-going research efforts are devoted to demonstrating *in vivo* performance in laboratory animals [35].

Finally, a patent (US 8750961 B1) with a multi-axis, multi-purpose sensor for use with implantable medical devices, and for simultaneously detecting the patient's posture and activity level has been developed [36]. The sensor includes a hermetically sealed, fluid-tight, bio-compatible housing. The housing is formed of a plurality of adjacently secured sides, and a plurality of side electrodes coupled to the sides. A central electrode is disposed at the geometric center of symmetry of the housing, to allow measurement of voltage changes between the central electrode and the side electrodes. A non-toxic electrically conductive electrolyte fills about half the housing, and immerses part of the central electrode and the side electrodes. The sensor further includes a low frequency bandpass filter for passing low frequency signals indicative of the patient's posture, and a high frequency bandpass filter for passing high frequency signals indicative of the patient's activity.

### 2.2. Research Challenges in Implantable Devices

**Figure 1.**
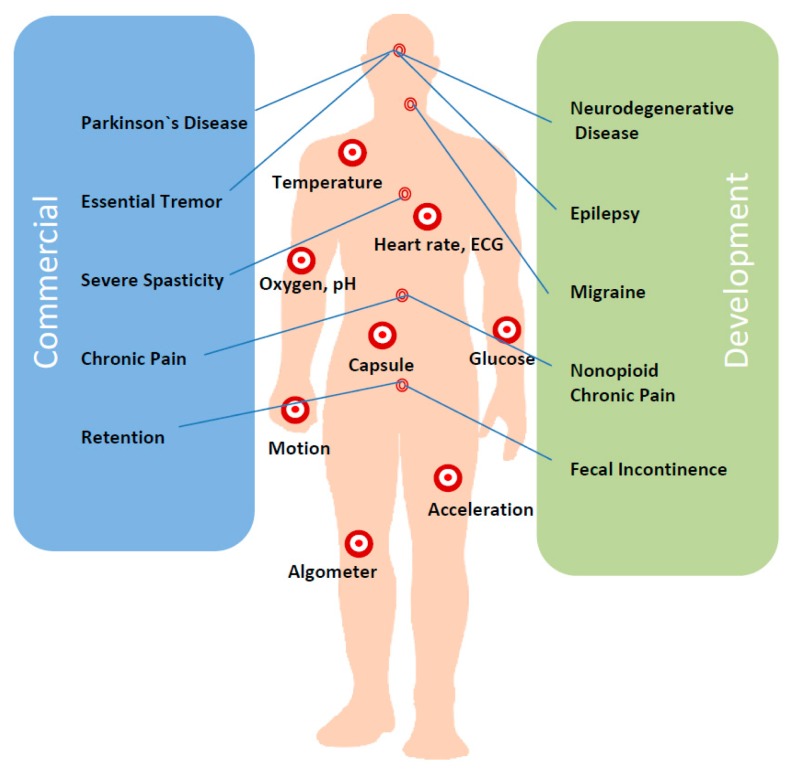
Mapping of implantable devices (based on [16]).

The new generation of implantable devices must overcome some main barriers at its conception stage as for example: size, energy available, power dissipation, power management, signal processing, communication of the measured data, bio-compatibility, chip-level integration, packaging, bioethics, and biosecurity [37]. A conceptual body map of commercial and in development phase implantable sensors is depicted in Figure 1, focussed on the most relevant disease processes based on Oesterle *et al.* [16].

There are very interesting implementations that combine ASICs, MEMS, and the design of integrated antennas for the RF powering of the system and the telemetry of the data, based on a link between external and internal coils. A fully wireless implantable cardiovascular pressure sensing system was developed by Chow *et al.* [38], combining a 130-nm technology and a MEMS capacitive sensor, powering the system through an external 35-dB-m RF powering at 3.7-GHz, with a distance range up to 10 cm inside the body, with a telemetry capability of 42.2 kb/s of channel data-rate, operating at 2.4-GHz and with a medical stent of 3 cm long. Cleven *et al.* [39] have also developed another interesting application regarding cardiovascular problems, where an implantable wireless system for monitoring hypertension is presented. The capacitive sensor, which is based on a MEMS implementation in a metal capsule, and the electronics (ASIC), forms a tip of 20 cm that will be placed in the femoral artery. In this case, the telemetry and powering is fixed at 133-kHz, with a maximum distance of 10 cm.

In Majerus *et al.* [40], a bladder-pressure-sensing implantable for chronic patients is introduced, based on a specific ASIC design, and also based on a RF powering (LC coupling at 3-MHz), and telemetry solution, but operating in an unlicensed ISM band (27.12-MHz), and a rechargeable battery solution. In this case, the size is also based on medical constraints given by minimally invasive cystoscopic surgery, defining a final capsule of 7-mm wide by 4-mm thick by 15-mm long. Another interesting implementation is related to glaucoma [41]. In this case, an RF intraocular pressure monitor is implemented based also in a MEMS solution for the sensor and in RF wireless transmission at 2.4-GHz and RF powering operating at 3.65-GHZ. In this way, RF powering and telemetry path are isolated in the same way as in the other examples. An additional approach is presented in Pivonka *et al.* [42], where RF powering and biomedical telemetry at 1.8-GHz are combined with the aim of developing a locomotive implantable.

Other interesting examples for close-loop systems are presented in [17,43,44]. Salam *et al.* [43] developed an implantable drug delivery system for the treatment of refractory epilepsy. The system is able to acquire real-time epileptic detection with focal antiepileptic drug injection feedback, combining electronics, pumps, reservoirs, *etc.* Lee *et al.* [17] presented an implantable microstimulator applied in a closed-loop cardiac pacemaker. Monge *et al.* [44] reported a fully intraocular epiretinal prosthesis, based on a 65 nm CMOS ASIC with 512 independent channels, integrated with a flexible MEMS origami coils, for the inductive powering at 10-MHz, and telemetry at 160-MHz, and parylene substrate to provide the intraocular implant.

**Figure 2.**
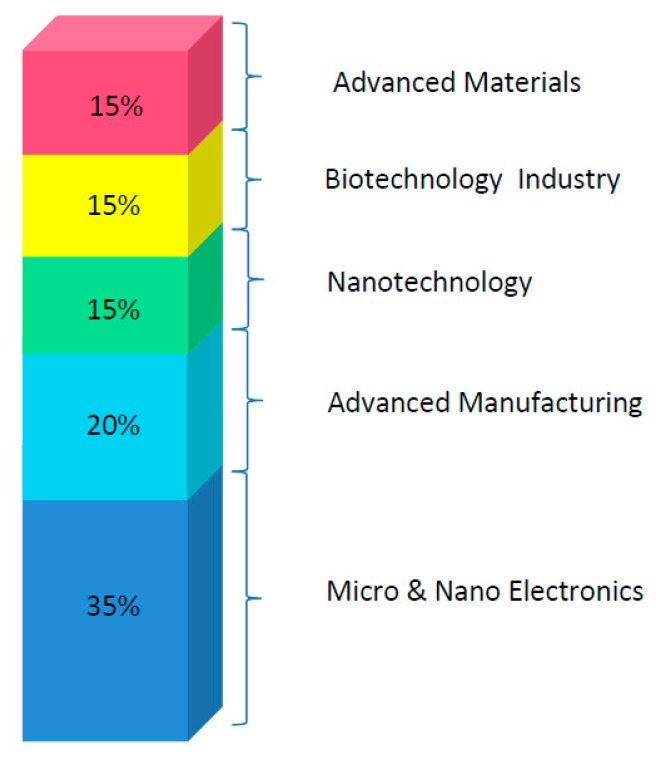
Suggested distribution of Key Enable Technologies (KETs) in a general implantable monitoring device.

Such complex systems are developed combining different Key Enabling Technologies (KETs) which are main contributors to overcoming the challenges involved in the development of an implantable device. Figure 2 introduces the suggested share of cross-cutting KETs involved in the development of a nano-enabled implantable device for *in vivo* biomarkers monitoring.

Current research is focused on the miniaturization and progression of the implants [45], in pursuit of less powering and long monitoring devices. The ongoing evolution in this field is based on the higher technological capabilities of microelectronic technologies, with higher density of integration, and involving a blend of MEMS, packaging and interconnects. The possibility of integrating new dedicated miniature transducers, such as pressure sensors [46], for arterial blood oxygen saturation, and accelerometers [47] for heart monitoring or cochlear implementations [48] are present examples. The progress in miniaturization of lab-on-chip solutions and integrated optics [49] opens the possibility of advances in new implantable medical devices and new challenges, culminating in the theranostic approach where the implantable will be able to deliver drugs. The system will implement the right algorithms for the control of drug delivery as well as the suitable reservoirs and pumps. This approach is feasible with the evolution and progress of MEMS, not particularly from silicon but from flexible polymers, and in terms of the lab-on-chip solutions in the field defined by medicine as micromachines [50], where there is an active control of fluids. The objective is to deliver drugs in a better way, more focalized in the local area or target of interest, rather than through traditional oral medication. The system should close the loop, with a monitoring and actuation role. The great paradigm is the artificial pancreas, with the design of an implantable system to monitor the glucose level, and a pump injector system. The combination of these enabling technologies creates the possibility of moving forward with advanced solutions such as artificial organs [50].

In the case of the optic approach, there is a significant limitation of integration, in terms of space, and also in terms of its implementation cost, but it is an interesting field of development. This approach has some important advantages compared with sensors based on an electrical measurable parameter, current or voltage, that make it interesting for integration in future developments. Among the benefits are its immunity to electromagnetic radiation, temperature tolerances, fewer risks to biological tissues and reliability when working in aqueous solutions. An interesting example is presented by Bingger *et al.* [46], where MEMS and an optoelectronic solution are implemented for continuous long-term monitoring of vital medical parameters such as arterial blood oxygen saturation, pulse and respiratory frequencies.

#### 2.2.1. The Powering Module

A main issue is the way the implant is powered. There are different options, always determined by the location of the packaged system, and the available area or volume location for the implantable. The first option is to feed the system through a battery [37]. In the field of battery-oriented biomedical and implantable devices [51], the classical approach is based on lithium batteries size C or D, summarized in Table 1.

**Figure 3.**
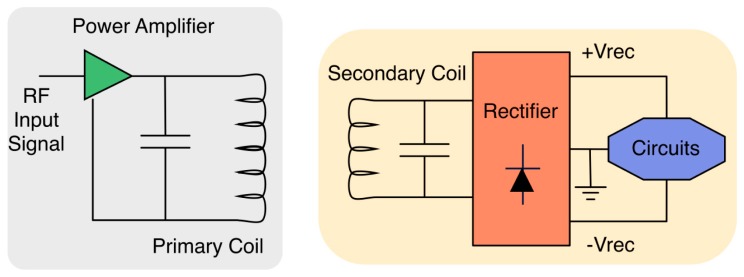
Generic wireless powering of an implantable device.

Current implementations of communication links in implantable devices are not suitable for many applications because of their poor harvesting efficiency [52]. Energy harvesting solutions must explore if a battery is not an affordable solution: these are defined as self-powered solutions [53]. The first solution could be based on electromagnetic induction [54], with various approaches. Some solutions propose the implementation of coils on a PCB substrate [55], or coaxial aligned coils with or without a ferrite rod [56], with all the attendant problems related to misalignments between the primary powering coil and the implanted secondary coil [57] and electronic implementations for a dynamic control of the power and voltage generated in the implantable in terms of the actual magnetic field that is generated [58]. On the one hand, we have an external element that plays the role of energy wireless power source, based on a class E amplifier, powered by the external battery, which supplies power to the implantable device through the skin [59,60] (see Figure 3). This powering is local, working for instance in the 13.56 MHz ISM (Industrial, Scientific, Medical) band [55], powering 10 mW at a distance of 10 mm between coils. The implantable operating at this ISM band must be placed near to the external generator. In Kilinc *et al.* [61], a particular case is presented. A wireless power-transfer *in vivo* implantable device for free moving small animals is derived. The scenario is that the living space for the animal is transformed into a full powering base.

However, this is one approach for the ISM band. The 13.56 MHz is a very low value. The more usual bands, taking into account the need to reduce the size of the implanted antennas and locations to place the implants in the body [62], are the bands of 433 MHz, which have similar results to the 402–405 MHZ MICS (Medical Implant Communication service) band [63], 915 MHZ, 2.45-GHz and 5.8-GHz. Currently, there are more works examining the optimization of the design of the implanted antennas [64] and the need to analyse the transmission losses between the external antenna and the deeply implanted antenna [65].

A particular example of this is introduced in Zhang *et al.* [66]. There, the rectifier module is designed to work at the 915-MHz ISM bandwidth (only in region 2). The performance of the RF source is quite small, from a typical 4 μW/cm^2^ for GSM to 1 μW/cm^2^ for the WiFi band. For coils, typical values are lower than 1 μW/cm^2^, but as much as 1 mW for close inductive coils (a few cm). In the 915-MHz ISM bandwidth, at 1.1 m, the energy recovered is around 20 μW [67]. New approaches are being developed. In particular, the use of ultrasonic powering instead of RF powering is of great interest. In Zhu *et al.* [68] and Moheimani *et al.* [69], 1 V is generated with a power capability of 21.4 nW.

Nevertheless, there are other approaches to power an implantable device without the use of a battery. One approach is based on the vibration energy harvesting point of view and the use of MEMS [53]. In Abidin *et al.* [70], a MEMS piezoelectric generator is used to harvest energy from vibrations; it also uses supercapacitors as storage elements. An example of a MEMS designed for implantable devices is given in Martinez-Quijada and Chowdhury [71], where it is stated that the micro-generator is able to generate more energy per unit volume than conventional batteries; that is, an RMS power of 390 μW for 1 mm^2^ of footprint area and a thickness of 500 μm, which is smaller than the volume of a typical battery in a pacemaker. Another approach is based on the use of fuel cells. The conception of a fuel cell as a biogenerator for implantable devices has emerged, with interesting results like in Zebda *et al.* [72], where a primary glucose fuel cell is derived for an implantable device. In some ways, the basic concept is the use of fluids in the body as a fuel source for the fuel cell, which would be an inexhaustible energy source. An interesting approach is the use of glucose as a fuel source, or the oxygen dissolved in blood [73,74]. Advanced approaches also explore a shift to the use of white blood cell capacities in biofuel cells [75]; or approaches such as that in Siu and Chiao [76], where the fuel cell is based on the use of a microorganism to convert the chemical energy of glucose into electrical energy, in a PDMS structure.

#### 2.2.2. The Encapsulation of the System

Bio-compatibility of the final device is a main barrier and challenge for the spread of implantable sensors. The encapsulation has to satisfy different properties, especially with regard to its lifetime. For instance, it has to be biocompatible and have a low dielectric constant [77], as well as being conformal and resistant.

Implantation of synthetic medical devices generates an immediate and complex material-related inflammatory response, such as blood and tissue incompatibility and bio-fouling [78]. Biofouling of the sensor membrane is an important cause of sensor dysfunction [79]. Therefore, the design of implantable BioMEMS devices must reduce this immune impact, minimize bio-fouling, reduce the physical effect of the implant on the surrounding tissues and reduce the degree of cell adhesion achieved by the implanted device. To avoid these adverse physiological effects, the implanted devices must be packaged with bio-compatible materials. However, bio-compatible materials might not always be compatible with the device requirements [78].

Currently, common and widely used materials in implanted biomedical devices with high compatibility are polyethylene glycol (PEG), polydimethylsiloxane (PDMS), PTMO (poly tetramethylene oxide [78] and parylene-C [80]. Polymer coatings are used for glucose sensors as they reduce the diffusion of interferences to the sensor while simultaneously balancing glucose and oxygen diffusion to enable an adequate glucose response. They are durable, inert, and capable of tolerating harsh environments produced by the FBR. Commonly evaluated polymers are, Nafion, polyurethane, polyethylene glycol (PEG), and hydrogels. Nafion is a perfluorosulfonic acid-based polymer that has been implemented as a bio-compatible coating. Polyurethane (PU) has been used extensively as an outer membrane to act as a bio-compatible interface with the surrounding host tissue. Surface passivation with polyethylene glycol (PEG) has been a widely studied strategy for resisting bio-fouling [81]. Hydrogels have a modulus similar to subcutaneous tissue and absorb water readily allowing easy diffusion of analytes to a sensor.

*In vitro* analysis in an osmotic glucose sensor evaluated identified 15 potential candidate materials which are shown in Table 2 below [79].

Other biocompatible materials include collagen layer for encapsulation [81], or gold, silicon nitride, silicon dioxide and SU-8 for coating use, able to reduce biofouling [82]. Coating of silicon carbide for example, can be used to significantly reduce thrombus formation on the surface of the devices, especially if the device is exposed to blood [83]. Bouaidat *et al.* [84] have also mentioned the use of phosphorous glass (SiPOC) for cell adhesion in BioMEMS.

The application of NDGA-crosslinked collagen scaffolds is also a good method for enhancing the function and lifetime of implantable bio-sensors by minimizing the *in vivo* foreign body response. Ju *et al.* [85] have developed a 3D porous and bio-stable collagen scaffold for implantable glucose sensors. The scaffolds were fabricated around the sensors and crosslinked using nordihydroguaiaretic acid (NDGA) or glutaraldehyde (GA) to enhance physical and biological stability. Kim *et al.* [86] reported an implantable sensor for real-time monitoring of the changes in bladder volume with PDMS and parylene-C. They find that both can be used as safe coating materials for the implantable bladder volume sensor reported.

A novel polymer coating consisting of poly(lactic-co-glycolic) acid (PLGA) microsphere dispersed in poly(vinyl alcohol) (PVA) hydrogels was evaluated in combination with dummy sensors as a “smart” drug eluting bio-compatible coating for implantable biosensors to prevent the foreign body response, and thus enhance sensor performance *in vivo* [80]. Single or multiple electro-spun layers can be used to address mass-transport limiting and additional membranes for improving biocompatibility of implantable biosensors and other biomedical devices requiring analyte transport, especially the first generation implantable glucose biosensors [87].

In summary, packaging techniques used must assure a long-term stability and surgical risks must be avoided. To fulfil these requirements, available implants in the market typically use hermetic packaging in laser-welded enclosures [88]. Nevertheless, for the envisaged miniaturized implants, where cans and micro-lids are used, this solution takes too much space. In that case, implantable devices for sensing and therapeutic purposes with active regions fully exposed to the physiological environment are a great challenge [89]. New approaches based on thin-film coating solutions are in progress to overcome these problems [90,91]. In Xie *et al.* [90] a bilayer solution based on an atomic layer deposited (ALD) Al_2_O_3_ combined with Parylene C for long-term encapsulation is presented, and in Sutanto *et al.* [91], a packaging and non-hermetic encapsulation MEMS flip chip technology for implantable devices is developed.

#### 2.2.3. The Nano-Biosensor

Special attention must be focused on nanobiosensors [92]: they need to combine accuracy, reliability, precision, life span, manufacturing and scalability, as well as address wealth and environmental risks, in order to overcome technological and market bottlenecks. A nanobiosensor or nanosensor is generally defined as a nanometre size scale measurement system comprising a probe with a sensitive biological recognition element, or bio-receptor, a physicochemical detector component, and a transducer in between. Two types of nanosensors with potential medical applications are cantilever array sensors and nanotube/nanowire sensors and nanobiosensors, which can be used to test nanolitres or less of blood for a wide range of biomarkers.

**Figure 4.**
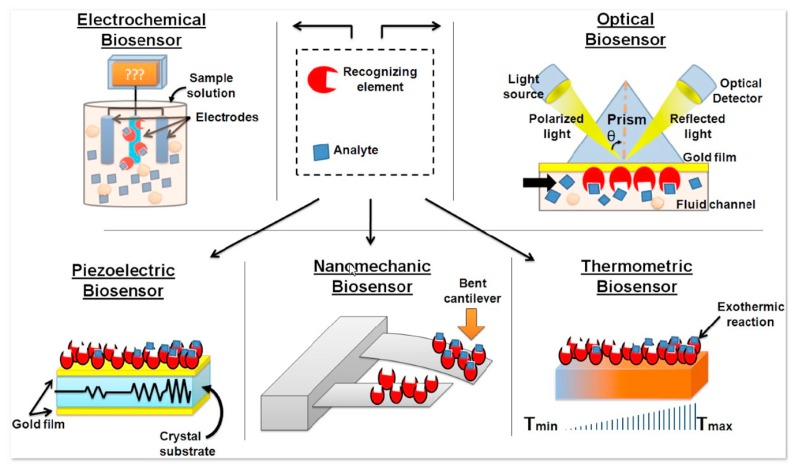
Generic function of several types of biosensor [93].

Then, a biosensor is a measurement system for the detection of an analyte that combines a biological component with a physicochemical detector. The general function of a biosensor is to convert binding events between biological receptors and target agents into a signal thanks to a transducer which can be based on an optical, a thermal, a gravimetric or an electrochemical detection (see Figure 4). This last category has gained increasing attention in the last few years. The high sensitivity, low cost and easy miniaturization of the electronic detection taken in conjunction with the wide range of applications, has resulted in these devices becoming a perfect analytical tool in different fields, such as diagnosis of genetic diseases, detection of infectious agents, study of genetic predisposition, development of personalized medicine, detection of differential genetic expression, drug screening, *etc.*

**Figure 5.**
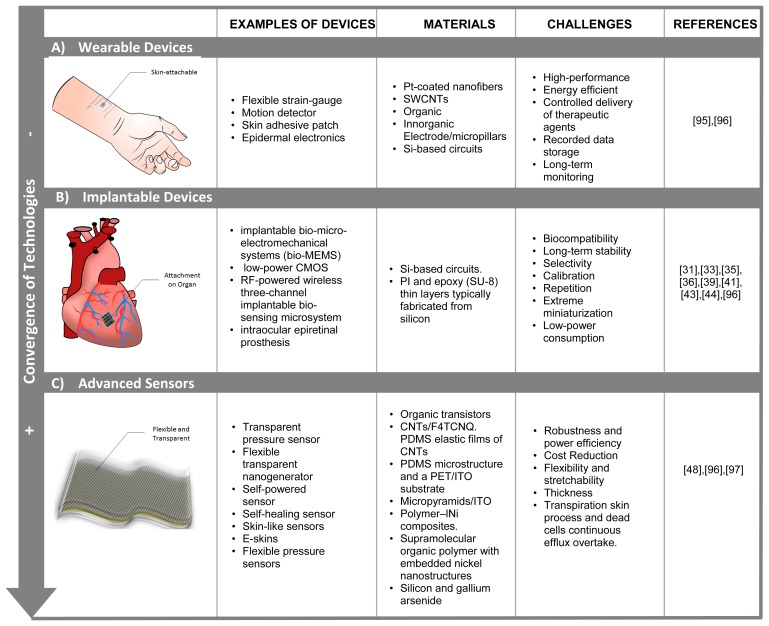
Summary of main devices for biomarkers monitoring.

The development of highly sensitive and low-cost sensors in the nanoscale, and its combination with nano-microfluidics solutions [94], based on micro-channels, micromixers and microvalves, are increasing the interest in the implementation of multi-parametric point-of-care devices, as a portable and low-cost solution to enhance diagnostics methods. In summary, Figure 5 shows principal technologies, challenges and materials for multipurpose implantable sensors.

## 3. Conception of the Bio-Implantable Customized Multi-Sensor

### 3.1. A Multipurpose Biosensor Architecture

Instead of defining a particular architecture of the implantable device for each sensor, the new approach in this paper introduces the design and use of a general architecture that will require minor modifications for a final customized implantable device which could be suitable for a set of specific applications.

**Figure 6.**
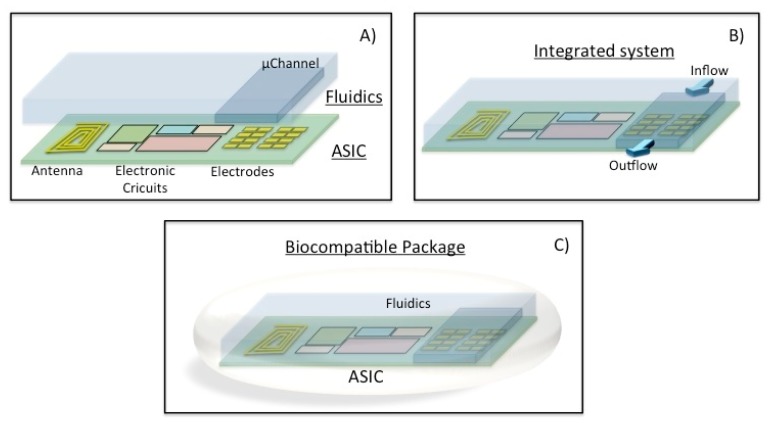
Schematic of the multi-parametric configurable implantable biochip system.

The objective is to have a generic array of nanosensors (electrodes) as an implantable system. Figure 6 shows the combination of cell clinic solutions concept as a lab-on-a-chip and electrical sensing techniques in a single implantable device.

**Figure 7.**
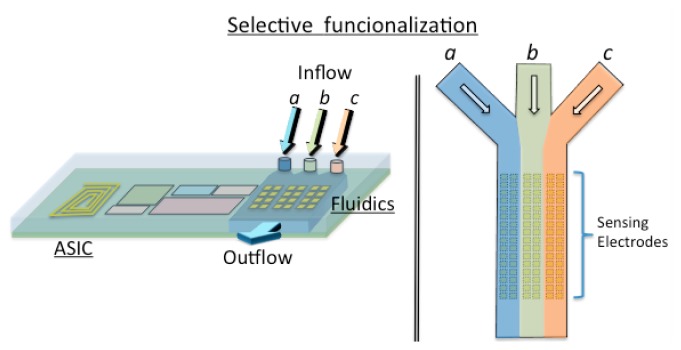
Microfluidic configurable array of biosensors on-chip.

The envisaged concept is applied in the definition of an on-chip configurable array of biosensors. This configuration will take place before the implantation thanks to a standard programmable bio-nano-chip approach [34]. A modular standard lab-on-a-chip approach will be followed to adapt the sensors in a quick, efficient and reliable way and then the implantable system will be placed into the patient (Figure 7). This concept of programmable platform could be adopted with the aim of developing a POC external device. These electrodes will be functionalized before the implantation in the human being thanks to the microfluidics (inflow/outflow) circuitry. Afterwards, the sensors will be checked and the chip cleaned and ready for the implantation.

The system will be enabled thanks to a system-on-a-chip (SoC) technology. CMOS microelectronics, MEMS and microfluidics will be combined to implement the programmed implantable, and easily adapted for the specific needs of the patient. The generic ASIC will combine the integrated electronics with an array of nano-biosensors (Sensors array), depicted as electrodes in Figure 6, which would be functionalized for particular purposes [98]. Generic modules for the power management, narcoleptic system design (NSD), communications, signal processing, the processor and data logging will be integrated to fulfil time-to-market constraints.

Tsai *et al.* [99] addressed the concept of the envisaged integrated multi-analyte biochip for an implantable device, in terms of the fabrication, where microfluidics (PDMS micro-channel), and a dielectrophoresis concentrator (DEP) are combined with external discrete electronics. The aim of the microfluidic system is to prepare and transport the fluid into the microcapillaries. Then, the preparation step consists in the separation of the fluidic and/or suspended particles [100], the mixing of the fluids for cell activation and mixing reactants for initiation. It could take place along the capillaries or inside of created droplets. These droplets are also useful to encapsulate biological particles or chemical reagents. In some cases, the sample also needs to be focalized [101] before it flows through the electrical or optical detection system as seen in Figure 7.

Based on the concept of Tsai *et al.* [99], the implantable multi-purpose sensor will be defined by the combination of configurable sensors as, for instance, glucose sensor [102,103], thermal metabolic sensor [104], PH and other sensors to detect the concentration of molecules, typically metabolites, such as glucose, lactose, sodium or ATP as examples of endogenous molecules, or exogenous molecules, such as etoposide and ifosfamide.

### 3.2. The Electronic Design

**Figure 8.**
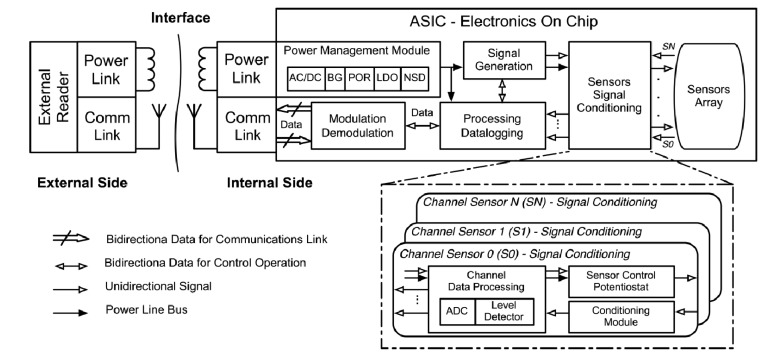
Bloc diagram of the proposed implantable architecture.

The envisaged integrated electronics is depicted in Figure 8. The ASIC will combine all the necessary electronic modules with the sensors' array of the functionalized biosensors. When the implantable is placed in the body, a programmed check of the state of the biosensors should take place. The system will check the sensors' array during the implantable life, and send a critical message to the final user if a malfunction is detected and the implantable must be removed while it is implanted.

The system will be based on the use of two different antennae, but it could be based on just one: one will be working at a lower frequency to harvest energy (power link), based on the previously presented concept of inductive powering, and a second antenna operating at higher frequencies for the communications (communications link). In this case, the communication link can be established around hundreds of MHz (usually in the 400 MHz ISM band) allowing higher communication rates and reducing the size of the antenna, as previously stated. The first antenna is focused to power the electronics through a dedicated inductive link operating at lower frequencies than the communication antenna. In that way, each antenna can be optimized for its functionality.

It is also possible to use just an inductive link for both purposes, and bi-directionally transmit the data [57]. However, the amount of transmitted information is limited and the size of the antenna is considerably bigger. The communication set-up could be based on a simple backscattering, defining an AM modulation, which is the approach taken. In our first ASIC implementation an inductive link for both purposes, operating at 13.56 MHz, was implemented. This is a good value for low power emission and appropriate to a subcutaneous placement. In our design a planar rectangular coil of 5.5 mm × 14.5 mm with a thickness of 0.5 mm has been designed, as a proof of concept for the antenna. It has seven turns with a conductor width of 0.2 mm. The design presents an inductance of 400 nH and a series resistance of 340 mΩ.

An AC/DC integrated rectifier generates an unregulated DC voltage from the electromagnetic energy delivered through the inductor link in the Power Management Module. The AC/DC block is based on a half-bridge NMOS rectified with a bulk control voltage.

The system has a power-on-reset module (POR) that activates the electronics when enough energy has been recovered through the inductive coupling. A LDO and a low-voltage low-power band gap reference circuit generate a DC regulated voltage to drive all the on-chip electronics. A NSD module is also implemented to enable the different modules thanks to the POR and the BG. The combination of these modules defines the Power Management Module.

Afterwards, the integrated electronics is introduced to drive the biosensor, make the measurement and to generate the data to be transmitted (Sensors Signal Conditioning). Usually, a low-voltage, low-power potentiostat circuit or similar instrumentations are used to control each sensor of the array (Sensor Control Potentiostat). CMOS electronics will be implemented for each of the sensor's array (Chanel Sensor) [105–107], combining different sensing techniques, such as chronoampetometry (CA) and cyclic voltammetry (CV), for sensors' characterization and calibration tasks, or electro-chemical impedance spectroscopy (EIS) generated by the Signal Generation Module, which will have the capability to generate DC voltages, a DC sweep or AC signal in order to cover the different techniques. In this case, we focus our attention on DC internal voltages which are designed to fix a DC voltage for the sensor. Three internal voltages of 0.6 V, −0.6 V, and 0.5 V can be selected. These signals are generated from the regulation module, based on the implemented band gap reference circuit. In our case, for a three electrode case, a low-voltage low-power CMOS potentiostat amplifier was implemented for an amperometric measurement.

These voltage levels could be applied by the potentiostat amplifier to the three electrode biosensor, defined by: (a) the working electrode (W), which serves as a surface on which the electrochemical reaction takes place and will be functionalized by the lab-on-a-chip module depicted in Figure 6 and Figure 7; (b) the reference electrode (R), which measures the potential at the W electrode; and (c) the auxiliary or counter electrode (A/C), which supplies the current required for the electrochemical reaction at the W electrode. A single potentiostat amplifier occupies an area of 327 μm × 260 μm, and has an average power consumption of 51.2 μW, which is smaller than Paglinawan [108] which has an area of 0.16 mm^2^, and a power dissipation of 600 μW, or Ahmadi and Jullien [109] which has a power dissipation greater than 150 μW. Its open-loop gain is 60 dB at low frequencies, and 50 dB1 kHz.

The current that is generated in the amperometric sensor, which is proportional to the electrochemical reaction that is generated at the working electrode, is measured by a transimpedance amplifier (TIA). Its input resistance of the design is 1 GΩ@DC, allowing a current detection up to 1 nA. The current-to-voltage conversion is defined as V_TIA_ = −I_W_ R_TRANS_, where I_W_ is the current through the working electrode and R_TRANS_ is the externally selected gain resistance. A second gain stage based on an inverter configuration follows the TIA and adapts the voltage values for the next stage, defining the Sensor Conditioning module.

The measured signal is forwarded to the *Data and Modulation Processing* modules. In this case a simple absence/presence detector is defined in the Chanel Sensor Module. The detection is based on the conception of an event-detector and the True/False detector works as an alarm: when the analyzed concentration level exceeds, under or over, a threshold value or the system detects the alarm condition, then the modulation process is activated to send the information to the external reader using a backscattering method through the inductive link, which can be AC or DC modulation.

### 3.3. Results

**Figure 9.**
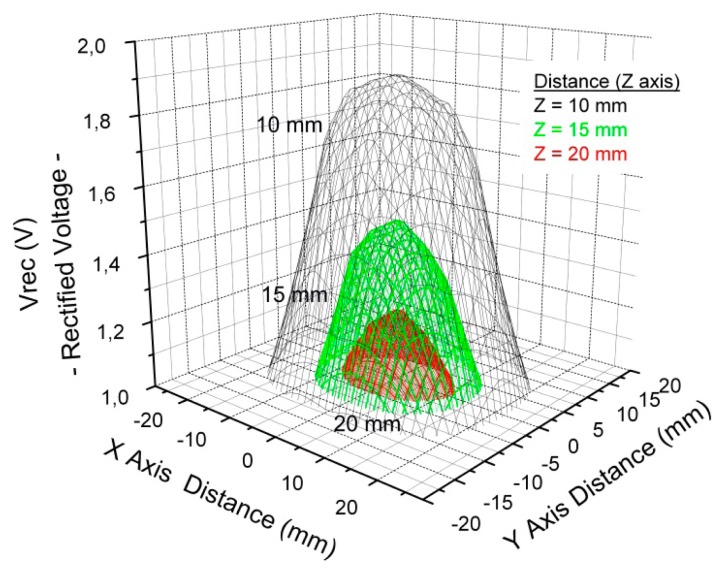
Distribution of the Vrec voltage in the XY plane for three different distances: 10, 15 and 20 mm (Reproduced from [60] with kind permission from Springer Science + Bussiness Media B.V).

A bipolar power scheme able to supply a regulated differential voltage of ±1.2 V and a maximum current of ±1.5 mA has been implemented. The Texas Instrument^®^ TRF7960 is used as external reader with a maximum emission power of 200 mW at 13.56 MHz. The desired on-chip regulated voltages of ±1.2 V are obtained for a distance up to 20 mm on air between coupling antennae. This analysis has been carried out in terms of the distance (Z-axis), between the external antenna and the coil designed in the PCB which defines the full implantable, that is, between the reader and the implantable. However, it is also necessary to have an approach to the misalignments between both antennas in the XY plane. Figure 9 depicts the rectified voltage (V_rec_) distribution in function of the XY misalignment for Z distances of 10, 15, and 20 mm. It can be noticed that the further the antenna is placed from the centre the lower the rectified voltage is. A more accurate study with human tissue is beyond the scope of this study.

A suitable solution for the detection of threshold values is based on the use of comparators, in terms of silicon area and power consumption, to detect one or several threshold values with medical interest. In the implemented approach, some comparators capable of detecting three different threshold voltages (V_th1_, V_th2_ and V_th3_), generated on-chip, have been implemented. These values are used to define a simple AM modulation protocol.

The signal is always a high level “1” but when a threshold value is achieved then a “0” level is generated. This functionality is based on the use of the comparators, monostables flip-flops and a very simple digital circuitry. As soon as there is enough voltage, the Power-On-Reset module generates a signal that activates the circuitry and the antenna starts to transmit continuously a series of “1”. When the first threshold level is achieved, the system transmits one zero (T_th1_). If the second is reached, two zeros are transmitted (T_th2_), and when the third is achieved a series of three zeros are sent (T_th3_). A zero time slot interval is defined as 250 ms (T_th1_ = 250 ms). In this way, the external reader can be quickly advised every time the desired substance exceeds the programmed threshold level or levels.

**Figure 10.**
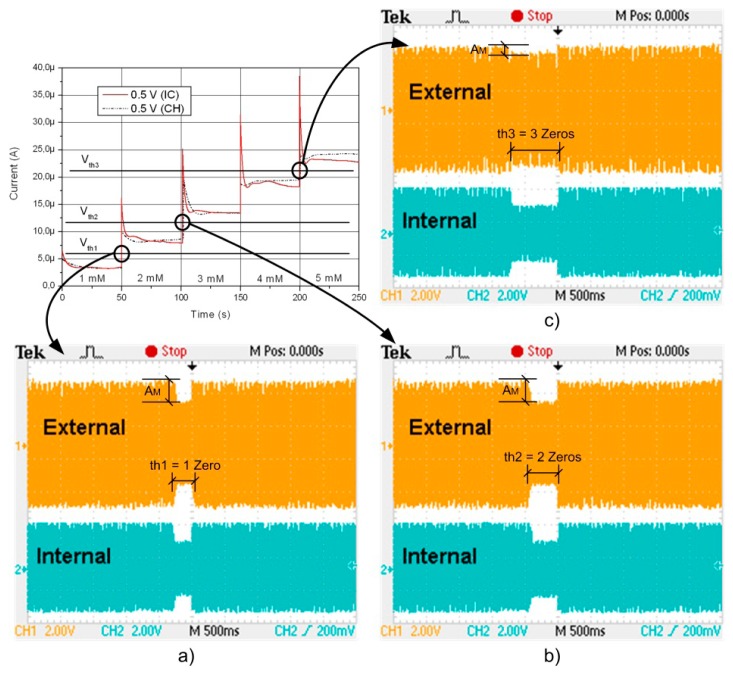
Amperometries measured with the prototype with three current levels programmed (Reproduced from [60] with kind permission from Springer Science + Bussiness Media B.V).

The instrumentation and the communication protocol were validated using several concentrations of K_4_[Fe(CN)]_6_ in PBS. In this case, a commercial sensor was used [60]. Several cyclic voltammetries (CVs) were carried out in order to verify the performance of the Control and conditioning modules. These measurements were compared with those obtained with a commercial potentiostat amplifier, the CH 1232A from CHInstruments®. These measurements were also used in order to calibrate the setup, and check the obtained values of the measured current peaks for the oxidation peak (around 240 mV), and the reduction peak (around 170 mV), for each concentration of K_4_[Fe(CN)]_6_ in PBS tested, from 1–5 mM. Also, this setup was used to validate the measured CV shapes obtained by the commercial equipment and the full-custom implementation. After the CV characterization some amperometric tests were done for different concentrations of K_4_[Fe(CN)]_6_ in PBS: 1 mM, 2 mM, 3 mM, 4 mM and finally 5 mM. Then, an experiment was carried out where the concentration was changed from 1 to 5 mM in time, with a fixed voltage of 500 mV in the sensor. This voltage is defined not at the oxidation peak. For this value of voltage applied in the sensor, the current varies from an average current of 3 μA (1 mM), to 16 μA (3 mM), up to 28 μA (5 mM). This experience was then carried out to validate the detection protocol, for a particular case implemented based on three threshold values. These values were programmed to detect the variations in the concentrations, defined by: Vth1, Vth2, Vth3, as is depicted in Figure 10, taking into account the current expected for each concentration case and defining different windows of comparison. When the first threshold is detected, then a first zero is transmitted, with a programmed width of 250 ms. When the second threshold level is reached, then two zeros are transmitted, in this case with an amplitude of 500 ms. Finally, in the particular case that a threshold Vth3 is defined to detect the highest concentration level, the modulation and data processing module will generate the longest transmission of zeros, in this case, three zeros with a total width of 750 ms.

## 4. Market Approach and Discussion

### 4.1. Innovation and Commercialization Chances in a Multi-KETs Scenario

In September 2009, the European Commission published its communication “*Preparing for our future: Developing a common strategy for key enabling technologies in the EU*” [110]. This strategy identifies the need for the EU to facilitate the industrial deployment of KETs in order to make its industries more innovative and globally competitive. KETs are one of the key factors in realizing the overall policy objectives of Europe 2020, due to the importance of these technologies for the competitiveness and innovation of European enterprises as well as for the development of sustainable products and processes [111]. In this context, Horizon 2020, the biggest Framework for Research and Innovation, has scheduled over 74 billion € for research funding focused on three fundamental pillars: 24.598 million € intended for Scientific Excellence, 31.748 million € for Society Challenges and 17.938 million € for Industrial Leadership. The last one aims to support SMEs in the industrial development and application of *KETs*, considered crucial accelerators for innovation and competitiveness [112].

KETs have been selected according to economic criteria, capital intensity, technology intensity, and their value adding enabling role [113]. The six KETs are: Nanotechnology, Micro and Nano Electronics, Photonics, Advanced Materials, Biotechnology Industry and Advanced Manufacturing Systems [114]. Among them, Nanotechnology is one of the most promising KETs due to its economic and social growth potential, since it has been considered the greatest impulse to technological and industrial development in the 21st century and the resource for the next industrial revolution [115–118].

**Figure 11.**
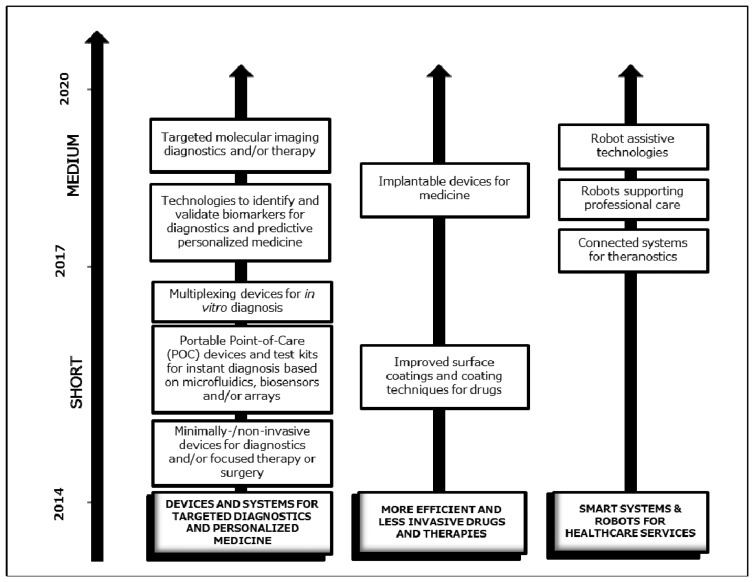
Fields for cross-cutting KETS developments in the Health and Healthcare Domain [113].

The integration of different Key Enabling Technologies (KETs) represents a vital activity in H2020. About one third of the budget assigned to KETs will go to supporting innovation projects integrating different KETs [119]. Cross-cutting KETs activities will in general include activities closer to market and applications. The global market volume in KETS are 646 billion euros and substantial growth expected is approx. 8% of EU GDP by 2015 [113]. In the Healthcare domain, short (2017) and medium (2020) perspectives of cross cutting KETs are shown in the Figure 11.

The European Commission stated that the EU has very good research and development capacities in some key enabling technology areas, but it has not been as successful in translating these results into commercialized manufactured goods and services [110]. R&D projects implemented in FP6 and FP7 frameworks have successfully delivered a lot of new nanomedicines but few products onto the market. In this context, the Commission states that bridging the so called “Valley of Death” to upscale new KET technology based prototypes to commercial manufacturing, often constitutes a weak link in the successful use of KETs potential. This is meant to be the “European Industrial Renaissance” by covering the whole value chain from Lab-to-Market as the principal aim of H2020 [113].

### 4.2. Market Forecast

The emerging sector of applied nanotechnology is addressed to biomedicine (nanobiotechnology and nanomedicine) which is the area of greatest projection of the future [120]. There are currently 247 nanomedical products that have been approved or that are in several stages of clinical trials. Industry market reports describing companies and their products related to nanomedicine and nanobiotechnology have also increased in the last several years [121]. It is expected that the annual global market for nano-related goods and services will top $3 trillion in 2020 [122]. Beyond, the medical sensors global market is expected to reach 15.5 USD billion in 2019, growing at a Compound Annual Growth rate (CAGR) of 6.3% from 2013 to 2019 [123]. Findings suggest that market growth for biosensors and biochips is virtually exploding. There are markets for biosensing technologies in the Asia-Pacific region, which show Compound Annual Growth Rates of 11% (2008–2018). Growth Rates of 10.7% occur in the highly developed market of the United States (US). In fact, this market is projected to reach $8.5 billion in US currency within five years, in about 2018 [124]. On the other hand, the global market for theranostic nanomaterial was valued at $112 billion in 2012 and is expected to reach $188 billion by 2017, registering a five-year CAGR of 10.8% for the period 2012–2017 [125].

Today, the implantable medical device market is oriented to the increasing elderly population and the associated increase in the prevalence of chronic degenerative diseases. However, the use of microtechnologies and MEMS in implantable devices is still in its infancy with few technologies currently approved for marketing in the US [126]. There is no identifiable market in the private sector for personalized and precision medicine yet [127]. The Food and Drug Administration (FDA) regulatory process will determine the concrete translation from benchtop research to commercialization of implantable nanosensors through clear and reasonable regulations. In this context, the FDA is collaborating with the interagency National Nanotechnology Initiative (NNI) to help formulate its guidelines with respect to many aspects of nanotechnology in the realms of cosmetics, diagnostics, and therapeutics [128].

### 4.3. Ethics Concerns

Designers of implantable medical devices have balanced safety, complexity, power consumption, and cost. However, today there are new concerns to take into consideration: security and data privacy [1]. As biosensors monitoring involves collection of data about vital body parameters from different parts of the body and making decisions based on it, the information is of a personal nature and is required to be secure [5]*.* The reason is to protect patients from acts of theft or malice, especially as medical technology becomes increasingly connected with other systems via wireless communications or the Internet. Implantable medical devices, including pacemakers, cardiac defibrillators, insulin pumps, and neurostimulators feature wireless communication [129].

Susceptibility to security breaches could compromise performance safety and the privacy of patients [2]. Burleson *et al.* [1] stated that there are two types of vulnerability: privacy, in which patient data is exposed to an unauthorized party, and control, in which an unauthorized person gains control of the device's operation or even disables its therapeutic services.

There is a need to ensure the privacy and security of medical data [4]. Recent analyses of implantable medical devices have revealed several security and privacy vulnerabilities [1]. For example, wireless connectivity could compromise the confidentiality of transmitted data or send unauthorized commands to the device [129]. Privacy specifications seem to be vague [4], in fact medical devices vary widely with regard to security features because no specific security guidance or requirements have been promulgated by the FDA [2].

Privacy-preserving methods should be developed for the comfort of the people monitored [3] and ensure reliable, secure communication and continued functionality while preserving patients' safety, confidentiality, and data integrity. There is nearly universal agreement on the importance of security for personal health information and electronic health records, but there is still a disagreement over the security requirements for medical devices [2].

Security must be considered in early design phases [1]. Some approaches have explored the feasibility of protecting an implantable device from privacy attacks by implementing security mechanisms entirely on an external device [129] or by encrypting data [1–3]. Moreover, in an effort to ensure security, personal authorizations and authentication have been proposed [20]. Therefore, Information and Communication Technologies (ICT) systems must facilitate the re-design of the current processes of care and follow up through the provision of services that enable the correct management of the patients within the healthcare organizations [130].

New and emerging technologies upset established moral norms by bringing to surface issues which were not previously open for discussion [131]. Argumentative patterns in this field are now known as NEST-Ethics (New & Emerging Science and Technology Ethics) [132].

## 5. Conclusions

After the revision of the current state-of-the-art of the implantable multi-sensor devices, the authors propose a generic multipurpose *in vivo* implantable biomedical device capable of detecting several threshold values for targeted concentrations. As a result, an integrated front-end architecture for *in vivo* customized detection is embedded within an implantable device with a generic array of nanosensors combining cell clinic solutions as a lab-on-a-chip and electrical sensing. The key point in this new conception is that, instead of defining a particular architecture of the implantable device for each sensor, the new approach introduces the design and use of a general architecture that will require minor modifications for the final customized implantable device that could be suitable for a set of specific applications.

Given the speed with which chronic diseases are increasing and the aging of the world population, the improvements that are possible with new theranostics techniques could have a great impact on the wellbeing and quality of life of the whole society while suitable biomedical devices are designed to reach a huge market over the next few years. Thus, a successful research, development, innovation and technology transfer may be fostered in a particular scenario typified by the convergence of technologies and disciplines, as well as by the combination of several KETs allowing the pilot lines and commercialization of cutting-edge devices embedding implantable sensors. Amongst all KETs, in this blending of technologies, nanotechnology seems to have a great impact, enabling new advantages in medical diagnostic or therapeutic devices, from the use of nanomaterials, in the development of nano-biosensors, by using the engineering of surfaces in order to improve the sensitivity of an electrode or its biocompatibility, and using nanoparticles from a therapeutic or diagnostic point of view, allowing modulation of treatment to particular targets within the human body, and ensuring delivery in an optimal way for a specific patient.

Although the case study reported in this paper is complex because it involves multiple organizations and sources of data, it contributes to extending experience to the most recent developments and practices on implantable sensors. The next step involves the development of a configurable application-specific integrated circuit (ASIC) working with a multiplexed array of nanobiosensors designed to be reactive for a set of target agents (enzymes, viruses, molecules, chemical elements, molecules, *etc.*). In this way, multiple sensors of the array can be used for one specific target, while other arrays can be prepared for the other targets, while also seeking a redundant response. As a result, a customized panel of biomarkers will be ready to be embedded into the bioimplantable medical device: each array will be used to detect a specific type of target, and the multiplexed system will be used to analyze each array focusing on a particular target. Then, top down approaches using nanoengineering and nanofabrication and bottom up approaches using supramolecular chemistry can produce novel diagnostics which will increasingly focus on delivering a personalized solution based on a real time analysis of array data, and where appropriate, applying this decision to deliver an automated therapy (theranostics).

The modular standard lab-on-a-chip approach introduced in this work may adapt the sensors in a quick, efficient and reliable way. Moreover, the system described in this paper must be tested before its implementation in a human being, and a POC platform would be designed for this purpose. The multi-parametric configurable implantable biochip system would be placed as a plug-and-play device. Moreover, it is needed to place a chip in the electronics module for the generation of the CV signals to check the sensors after their functionalization. Communications and powering will follow the same wireless approach as the implantable device. Once the performance of the sensors has been certified and cleaned-up, the implantable system will be suitable for being placed in the patient. This concept of programmable platform could be adopted in the design of a POC external device.

On the other hand, despite the somewhat limited availability of information discussing the safety of implantable sensors, the case study presented in this paper is a clear demonstration of how to take into account biocompatibility challenges and ethical concerns to foster the development of new bioimplantable medical devices. At this point, the bonds between the science community, hospitals, industry and citizens need to be strengthened with the aim of enhancing biomedical research on implantable sensors and its commercialization. Doubtless, biomedical devices represent a strategic gamble for the future of scientific and technological policy areas as they seek accelerated economic growth within the knowledge-based society and confront the new scientific and market challenges presented by the nano-enabled implantable biomedical devices.

Finally, the present and future of the implantable devices goes beyond these objectives and research challenges. There is a great transformation in medical diagnostics and the blend of the different KETs for the integration and commercialization of these devices should follow a standardization process to propel them in a Moore's Law trajectory as happened with the microelectronics revolution.

## Figures and Tables

**Table 1.** Types of batteries for biomedical devices (based on [51]).

**Cell Size**	**C**	**D**	**R11098**
Diameter (mm)	26.0	33.9	26.92
Height (mm)	50.5	61.5	42.93
Thickness (mm)	NA	NA	8.94
Weight (g)	65	15.5	28
Volume (cc)	26.8	55.5	10.33
Rated Capacity (Ah)	1.9	4.3	0.575

**Table 2.** Candidate materials for implementation in the glucose sensor [79].

	**Material**	**Abbreviation**	**Specification**	**Manufacturer**
Encapsulation Materials	Sylgard 184	PDMS	Polydimethylsiloxane	Dow Corning Corp., Midland, MI
	Araldite 2020	A2020	Epoxy resin	Huntsman, Duxford, UK
	Stainless steel	Me	Corrosion resistant, Type316L	Fosstech Engineering Stokke, Norway
Membrane Materials	Silicon	Si	Silicon with native 2–3 nm oxide surface	HiVe, Horten, Norway
	Silicon Dioxide	SiO_2_	Silicon with a 500 nm thick thermal oxidized surface	HiVe, Horten, Norway
	Cellulose ester	Cm	Ultrafiltration membrane (MWCO 5000 Da, - 2.5 nm	Spectrum Laboratories Europe B.V., Breda, Netherlands
	Polyamide	PATF	Thin Film membrane (MWCO 0 Da), <1 nm	Sterlitech Corporation, Kent, WA
	Polycarbonate	PC	Track-etched membrane (MWCO 500 kDa, - 15 nm)	Watman, Kent, UK
	Aluminum oxide	AAO	Anodic aluminum oxide (MWCO 50 kDa, - 5 nm)	Synkera Technologies, Longmont, CO
Sensor Carrier Materials	CeramTec GC	CT	Low temperature cofired ceramic (LTCC)	Ceramtec AG, Plochingen, Germany
	Dupont 951	DP	Low temperature cofired ceramic (LTCC)	Dupont, Wilmington, DE
Sealing Materials	Silicone 3140 coating	S3140	Silicone-based polymer	Dow Corning Corp., Midland, MI
	Silicone 3145 adhesive	S3145	Silicone-based polymer	Dow Corning Corp., Midland, MI
	Epo-Tek 353ND	ETek	Epoxy resin	Epoxy Technol., Billerica, MA
